# Native Potato Starch and Tara Gum as Polymeric Matrices to Obtain Iron-Loaded Microcapsules from Ovine and Bovine Erythrocytes

**DOI:** 10.3390/polym15193985

**Published:** 2023-10-04

**Authors:** Carlos A. Ligarda-Samanez, Elibet Moscoso-Moscoso, David Choque-Quispe, Betsy S. Ramos-Pacheco, José C. Arévalo-Quijano, Germán De la Cruz, Mary L. Huamán-Carrión, Uriel R. Quispe-Quezada, Edgar Gutiérrez-Gómez, Domingo J. Cabel-Moscoso, Mauricio Muñoz-Melgarejo, Wilber César Calsina Ponce

**Affiliations:** 1 Nutraceuticals and Biomaterials Research Group, Universidad Nacional José María Arguedas, Andahuaylas 03701, Peru; dchoque@unajma.edu.pe (D.C.-Q.); bsramos@unajma.edu.pe (B.S.R.-P.); huamancarrionmary@gmail.com (M.L.H.-C.); 2 Research Group in the Development of Advanced Materials for Water and Food Treatment, Universidad Nacional José María Arguedas, Andahuaylas 03701, Peru; 3 Department of Education and Humanities, Universidad Nacional José María Arguedas, Andahuaylas 03701, Peru; jcarevalo@unajma.edu.pe; 4 Agricultural Science Faculty, Universidad Nacional de San Cristobal de Huamanga, Ayacucho 05000, Peru; german.delacruz@unsch.edu.pe; 5 Agricultural and Forestry Business Engineering, Universidad Nacional Autónoma de Huanta, Ayacucho 05000, Peru; uquispe@unah.edu.pe; 6 Engineering and Management Faculty, Universidad Nacional Autónoma de Huanta, Ayacucho 05000, Peru; egutierrez@unah.edu.pe; 7 Ambiental Engineering, Universidad Nacional San Luis Gonzaga, Ica 11001, Peru; jesus.cabel@unica.edu.pe; 8 Human Medicine Faculty, Universidad Peruana los Andes, Huancayo 12006, Peru; d.mmunoz@upla.edu.pe; 9 Social Sciences Faculty, Universidad Nacional del Altiplano, Puno 21001, Peru; wcalsina@unap.edu.pe

**Keywords:** native potato starch, tara gum, microencapsulation, erythrocytes, iron released, spray drying

## Abstract

Iron deficiency leads to ferropenic anemia in humans. This study aimed to encapsulate iron-rich ovine and bovine erythrocytes using tara gum and native potato starch as matrices. Solutions containing 20% erythrocytes and different proportions of encapsulants (5, 10, and 20%) were used, followed by spray drying at 120 and 140 °C. Iron content in erythrocytes ranged between 2.24 and 2.52 mg of Fe/g; microcapsules ranged from 1.54 to 2.02 mg of Fe/g. Yields varied from 50.55 to 63.40%, and temperature and encapsulant proportion affected moisture and water activity. Various red hues, sizes, and shapes were observed in the microcapsules. SEM-EDS analysis revealed the surface presence of iron in microcapsules with openings on their exterior, along with a negative zeta potential. Thermal and infrared analyses confirmed core encapsulation within the matrices. Iron release varied between 92.30 and 93.13% at 120 min. Finally, the most effective treatments were those with higher encapsulant percentages and dried at elevated temperatures, which could enable their utilization in functional food fortification to combat anemia in developing countries.

## 1. Introduction

In recent years, new biopolymers have been studied for their use in encapsulating different bioactive compounds beneficial to human health [1,2]. The development of research on mixtures of matrices and cores is essential to understand their interactions using modern physical, chemical, and structural techniques [3,4]. The spray drying microencapsulation is used to stabilize various phytochemicals that are applied in the food and pharmaceutical industry [5,6]. The wall materials present in the microcapsules provide additional health benefits due to their protein and fiber content [7,8].

Iron is an essential micronutrient in several vital functions, such as oxygen transport, cell proliferation, immunity, deoxyribonucleic acid synthesis, and energy production. Heme iron is obtained from meat, offal, and blood containing myoglobin and hemoglobin. In contrast, non-heme iron is ferric (Fe^+3^) or ferrous (Fe^+2^) salt in cereals, dairy products, legumes, and other vegetables [9,10]. Iron deficiency causes anemia, mainly in children, due to the low consumption of foods containing this mineral. Heme iron is the most easily bioavailable form in the human organism, with between 15 and 40% absorption; current studies indicate that blood is one of the primary sources of iron [11,12].

Iron deficiency anemia is a global public health problem, and various iron fortification and supplementation strategies have been developed to improve its bioavailability and absorption [13]. Iron encapsulation is a promising technique that protects iron from oxidation and degradation reactions during food processing and incorporation. Iron can be explicitly released in the intestine through microencapsulation, improving absorption. Various materials have been used as encapsulants, including starches, maltodextrins, chitosan, and alginate [14]. Multiple encapsulation methods are applied in the food industry, such as emulsification, ionic gelation, extrusion, spray drying, and lyophilization [15,16,17,18,19]. In addition, novel chemical encapsulation techniques have been developed, such as interfacial polymerization, molecular co-crystallization, and inclusion in cyclodextrins [20,21]. Polymeric systems containing inorganic Mg and CaCO_3_ substances that promote biodegradation, biocompatibility, and bioactivity are also being developed. The controlled incorporation of these inorganic substances into the polymers modifies the mechanical, thermal, and biological properties that could also be used for iron [22,23].

Different drawbacks were identified in fortifying foods with iron, such as adverse changes in sensory characteristics, gastrointestinal discomfort, and using non-heme iron in higher proportions. These problems have been improved by using encapsulation technology, a micro-packaging process in which a wide variety of iron compounds are protected with different polymeric matrices producing microcapsules and nanocapsules via different methodologies, among which spray drying stands out. In this way, different forms of encapsulated iron have been obtained and tested for their effectiveness in vitro and in vivo, with promising results in reducing iron deficiency anemia [9,24,25]. This paper presents an innovative methodology for obtaining iron-loaded microcapsules from sheep and cattle erythrocytes using native potato starch and tara gum as polymeric matrices. These microcapsules offer a promising application in controlled iron release, which could have significant implications for developing treatments for anemia and other related diseases.

The development of controlled release systems for bioactive compounds has become highly relevant in medicine and nutrition. Iron is essential for proper body function, and its deficiency can lead to serious health problems. Encapsulating iron in microcapsules could improve its bioavailability and enable sustained release in the body, avoiding toxicity issues associated with high doses. In this study, the use of native potato starch and tara gum as polymeric matrices to encapsulate iron from sheep and cattle erythrocytes was explored, aiming to obtain microcapsules capable of releasing the mineral in a controlled manner.

## 2. Materials and Methods

### 2.1. Materials

The blood of sheep (*Ovis orientalis aries*) and cattle (*Bos taurus*) was collected at the Municipal Slaughterhouse in San Jerónimo, province of Andahuaylas, Peru, which the National Agrarian Health Service authorizes. The blood extraction was carried out under entirely safe and aseptic conditions. Local farmers from the district of Ocobamba, province of Chincheros, Peru, kindly provided the tara. The native potato of the yanapalta variety was acquired at the central market of the district of Andahuaylas and was in optimal conditions for consumption. The research involving the use of animals was approved by the Ethics Committee of the National University José María Arguedas through Resolution N° 232-2020-CO-UNAJMA dated 22 September 2020. Sheep and cattle blood was selected because these animals are abundant in the study area, and their blood is an iron-rich by-product that is not used during the processing of these animals. In the case of the native potato, the yanapalta variety was chosen because of its good yields in the field and as it is an excellent source of native starch. As for the tara, it was chosen because it is a rich source of gum that is found in abundance on the study site.

The reagents used were hydrochloric acid reagent grade (Spectrum Chemical Mfg. Corp., Bathurst, NB, Canada), nitric acid reagent grade (Spectrum Chemical Mfg. Corp., Bathurst, NB, Canada), and absolute ethanol (Scharlau, Sentmenat, Spain).

### 2.2. Native Potato Starch

Around 5 kg of native potatoes from yanapalta variety were used. These were crushed using a Bosch blender (Stuttgart, Germany). Following this, several rinses were carried out with distilled water to isolate the starch by allowing it to settle. The acquired starch was then dried at 50 °C using a FED 115 BINDER forced convection oven. Subsequently, it was finely ground into powder using an agate mortar. Next, the starch underwent sieving with an analytical vibrating sieve AS 200 (Retsch, Haan, Germany) with a 45 μm mesh size. The resultant starch was collected in airtight containers and stored at 20 °C for future use [10,26,27].

### 2.3. Tara Gum

The germ was isolated from tara seeds, and 30 g of the germs were mixed with 800 mL of distilled water. The mixture was then stirred for 12 h at 80 degrees Celsius. Afterward, the solution was filtered through a 150 µm nylon mesh screen. It was combined with 96% ethanol at a 1:1 ratio to purify and cause the gum to precipitate. Following this, the resulting gum was diluted with distilled water until it reached a viscosity of 30 cP using a viscometer (DV-E Brookfield Engineering Laboratories, Inc., Middleboro, MA, USA). The extract was spray-dried using a mini spray dryer B-290 from BÜCHI Labortechnik AG, operating at an inlet temperature of 100 °C and an airflow rate of 650 L per minute [10].

### 2.4. Spray-Dried Erythrocytes

Sodium citrate was utilized as a blood anticoagulant (at a concentration of 3 g/L) to collect blood samples from sheep and cattle. The obtained blood was centrifugated at 3000 revolutions per minute for 10 min (CR4000R Centurion, Pocklington, UK). This process aimed to separate the cellular components from the rest. The resultant pellet was rinsed twice with a saline solution containing 0.15 M NaCl. After completing the washing procedures, the viscosity of the solution was adjusted to reach 30 cP (DV-E Engineering Laboratories, Inc.). Next, the material was subjected to drying using a B-290 mini spray dryer from BÜCHI Labortechnik AG, operating at an inlet temperature of 120 °C and an airflow rate of 650 L/h. The resulting atomized material was collected within low-density polyethylene bags and subsequently stored in a desiccator at 20 °C until it was ready for subsequent utilization [10].

### 2.5. Erythrocyte Microparticles

For the microencapsulation process, native potato starch and tara gum at a 4:1 ratio were used as the outer layer materials. These components were prepared at varying concentrations of 5%, 10%, and 20% by weight/volume (*w*/*v*). The solution for encapsulation was prepared a day in advance. As far as erythrocytes were concerned, they were made at a constant concentration of 20% (*w*/*v*). Both solutions were mixed at a 1:1 ratio and blended thoroughly using an ultraturrax device (Daihan, model HG15D, Wonju, Republic of Korea), operating at 7000 revolutions per minute for 5 min. The actual encapsulation was carried out using a B-290 mini spray (BÜCHI Labortechnik AG, Flawil, Switzerland). This process occurred at inlet temperatures of 120 °C and 140 °C and an airflow rate of 650 L/h. Following this, the encapsulated materials were collected and carefully placed into low-density bags and stored within a desiccator at a temperature of 20 °C [10]. 

**Figure 1:**
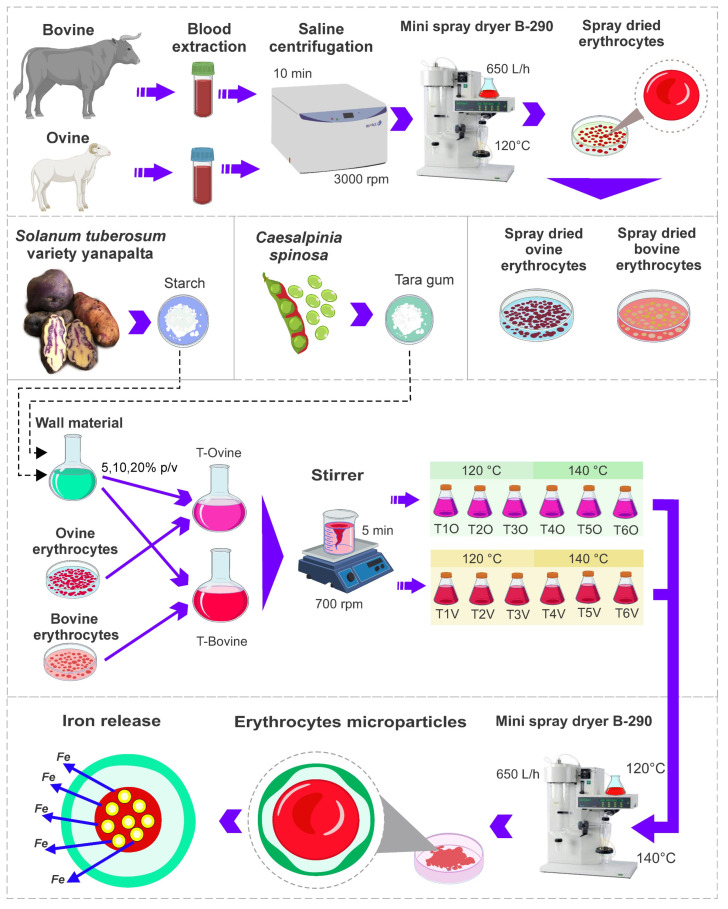
Experimental flow diagram.

The experimental flow diagram is shown in Figure 1, in which the abbreviations T1O, T2O, T3O, T4O, T5O, and T6O are presented to refer to the ovine erythrocyte microencapsulation treatments, and the abbreviations T1V, T2V, T3V, T4V, T5V, and T6V for the bovine erythrocyte microencapsulation treatments.

### 2.6. Iron Content

A total of 200 mg of the sample underwent treatment with 3 mL of HCl and 9 mL HNO_3_. The resulting mixture was then adjusted to a final volume of 50 mL using ultrapure water. The prepared solutions were then subjected to microwave digestion by utilizing a microwave digester (SCP Science, Miniwave, QC, Canada). An axial mode inductively coupled plasma optical emission spectrometer ICP-OES 9820 138 (Shimadzu, Kyoto, Japan) was employed to measure the iron content. Argon gas was maintained at a flow rate of 10 L/min during the measurements and readings were taken at a specific wavelength of 239.562 nm [10].

### 2.7. Yield, Moisture, Water Activity, and Bulk Density 

The yield was calculated by considering the mass of the obtained spray-dried powder and the initial mass (wall material and core) according to the following relationship [28]:

(1) $$Y\;\left(\%\right)=\frac{mi}{mf}\times 100\;$$

where $Y\;\left(\%\right)$ represents the encapsulation yield, mi is the initial mass (g), and mf is the final mass of the spray-dried powder (g).

Moisture content was determined following the AOAC 950.10 oven drying method [29]. The water activity was assessed using a water activity meter (Rotronic, Bassersdorf, Switzerland) [30]. Bulk density was calculated by dividing the mass of the microcapsules by the volume measured using a graduated 10 mL cylinder [28].

### 2.8. Color Analysis

Lightness L* and chroma a* and b* color attributes were ascertained using a benchtop colorimeter (CR-5, Konica Minolta, Tokyo, Japan). The degree of color change was computed using the subsequent equation [31]:

(2) $${\Delta E}_{ab}^{*}=\sqrt{{\Delta L}^{*2}+{\Delta a}^{*2}+{\Delta b}^{*2}}\;$$

where ${\Delta E}_{ab}^{*}$ is the color variation and ${\Delta L}^{*}$, ${\Delta a}^{*}$, and ${\Delta b}^{*}$ are the differences between L*, a*, and, b* initials and finals.

### 2.9. Amylose and Amylopectin Content

The potato amylose standard (Sigma Aldrich, St. Louis, MO, USA) was used in concentrations of 0.1 to 1.0 mg/mL for the calibration curve. For amylose extraction, 20 mg of sample was taken and 0.2 mL of 95% ethanol (Scharlau, Senmanat, Spain) and 1.8 mL of 1 M NaOH (Sigma Aldrich, Darmstadt, Germany) were added and allowed to stand for 24 h at room temperature. Subsequently, the volume was adjusted to 20 mL with ultrapure water and homogenized in a vortex at 2000 RPM for minutes. For the colorimetric reaction, 0.5 mL of the extracted solution, 1 mL of 1M acetic acid (Sigma Aldrich, St. Louis, MO, USA), and 0.2 mL of lugol solution were taken, and the volume was made up to 10 mL with ultrapure water. The solution was shaken and allowed to react for 20 min, protected from light. Absorbance readings were carried out at a wavelength of 620 nm using a UV spectrophotometer (CR-5, Konica Minolta, Tokyo, Japan) [32,33].

### 2.10. Total Organic Carbon

A total of 0.05 g of encapsulated samples was positioned within ceramic containers to be analyzed utilizing a total organic carbon analyze TOC-L CSN-SSM 5000A (Shimadzu, Kyoto, Japan) [34,35].

### 2.11. SEM-EDS Analysis

The structure of erythrocytes and microcapsules was examined using a scanning electron microscope (SEM Thermo Fisher, Waltham, MA, USA) under low vacuum conditions, employing an acceleration voltage of 25 kV. Furthermore, an energy-dispersive X-ray spectroscopy (EDS) detector was utilized to perform surface chemical analysis of the samples [36].

### 2.12. Particle Size and ζ Potential Analysis 

For particle size determination, a laser diffraction instrument, Mastersizer 3000 (Malvern Instruments, Worcestershire, UK) was employed. The samples were dissolved in isopropyl alcohol, subjected to sonication for 60 s, and measured at 600 nm. As for determining the ζ potential, 25 mg of erythrocytes and microcapsules were homogenized in 50 mL of ultra-pure water. The analysis was conducted using a dynamic light scattering (DLS) instrument, Zetasizer ZSU3100 (Malvern Instruments, Worcestershire, UK) [36].

### 2.13. FTIR Analysis

Fourier transform infrared spectroscopy (FTIR) was used to analyze and identify functional groups in erythrocytes and microcapsules. Pellets were prepared by combining 2 mg of the sample with 200 mg of KBr. The mixture was then pressed at a force of 10 tons to create the pellets for analysis. The FTIR measurements were conducted using the transmission module of the Nicolet IS50 FTIR (ThermoFisher, Waltham, MA, USA). The spectral range covered wavelengths from 4000 to 400 cm^−1^. Readings were taken with a scan repetition of 32 and a resolution of 8 cm^−1^ [37].

### 2.14. Thermal Analysis 

For the thermal stability analysis using Thermogravimetric Analysis (TGA), and 10 mg of erythrocytes and microcapsules were utilized. The measurements were conducted using a TGA 550 thermal analyzer (TA Instrument, New Castle, DE, USA) with a heating rate of 10 °C/min. Furthermore, a Differential Scanning Calorimeter (DSC2500, TA Instruments, New Castle, DE, USA) was utilized for analysis. In this process, 2 mg of microcapsules were employed. The temperature range covered was from 0 to 250 °C, employing a heating rate of 10 °C per minute. The analysis was conducted under a nitrogen atmosphere [37].

### 2.15. Iron Release 

To conduct the in vitro release determination, uniform solutions were prepared by mixing 0.05 g of microcapsules with 500 mL of a 0.1 N HCl solution. These prepared samples were then subjected to a water bath with an agitation system (WTB 50, Memmert, Schwabach, Germany) at a temperature of 37 °C. Extractions were carried out at specific intervals: 0, 30, 60, 90, and 120 min. After extraction, the samples were analyzed on an Inductively Coupled Plasma Optical Emission Spectrometer (ICP-OES) model 9820 138 (Shimadzu, Tokyo, Japan). The resulting data were quantified in mg of Fe/g of sample and calculated using the provided relationship [10]:

(3) $$\%L=\frac{{Fe}_{T}}{{Fe}_{o}}\times 100$$

where %L is the percentage of release, ${Fe}_{T}$ is the iron content at time t (mg/g), and ${Fe}_{o}$ is the initial iron content (mg/g).

### 2.16. Statistical Analysis

Data analysis was performed using the Origin Pro 2022 software (OriginLab Corporation, Northampton, MA, USA). The analysis involved the analysis of variance (ANOVA) along with Tukey’s multiple range test, employing a significance level of 5%.

## 3. Results and Discussions

### 3.1. Instrumental Characterization of Matrices and Cores

#### 3.1.1. SEM-EDS Analysis, Particle Size, ζ Potential, Color, and Iron Content

**Figure 2:**
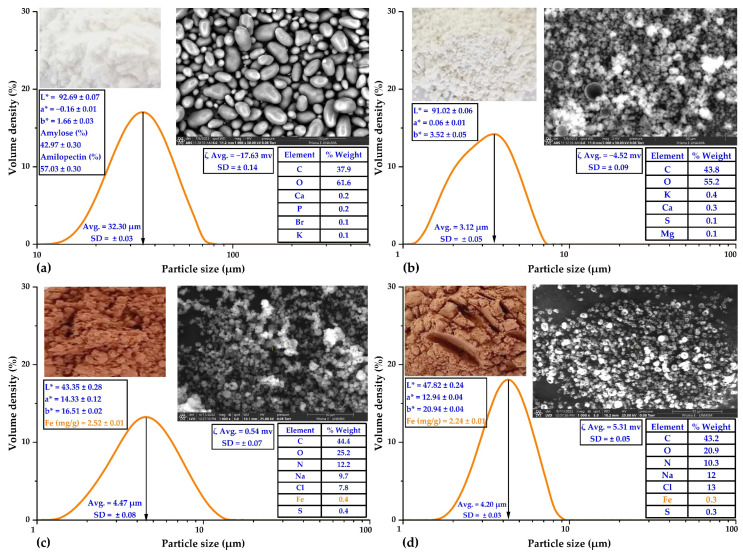
(**a**) Characterization of native potato starch of the yanapalta variety, (**b**) characterization of spray-dried tara gum, (**c**) characterization of spray-dried ovine erythrocytes, and (**d**) characterization of spray-dried bovine erythrocytes.

In Figure 2a, the characterization of native potato starch from the yanapalta variety is shown, where elliptical shapes of granules were observed with a size of approximately 32.30 µm, with a negative ζ potential, white color, and a predominant presence of carbon and oxygen on its surface, these results were similar to those reported for starch from native potatoes of the peruanita variety [36]. Regarding specifically the SEM-EDS analysis, the results were similar to the starches obtained from native potatoes grown in Cusco, Peru, which presented smooth surfaces and ellipsoidal shapes that also contained mainly carbon, oxygen, and traces of calcium, with particle sizes between 12 and 72 µm [38]. The above parameters are essential in the chemical and techno-functional properties of starch [39]. Amylose (42.97%) and amylopectin (57.03%) were also characterized, which is consistent with native potatoes of the huamantanga and qeccorani varieties [40]. The ratio of amylose to amylopectin influences the functional properties of starches [41]. 

In Figure 2b, spray-dried tara gum is characterized by spherical particles obtained with an average size of 3.12 µm, negative ζ potential, white color, and a predominant presence of carbon and hydrogen on its surface. In this context, recent investigations support the notion that spray drying enables the production of spherical and uniformly sized particles [37]. The negative zeta potential could be attributed to carboxyl and hydroxyl functional groups on the particle surface, which are crucial in stabilizing colloidal dispersions [10]. The white color of the microparticles could be attributed to their size and morphology, influencing the dispersion of visible light; the predominant presence of carbon and hydrogen on the surface is likely associated with the chemical structure of tara gum, which is rich in polysaccharides [10,36].

On the other hand, Figure 2c,d depict the instrumental characterization of ovine and bovine erythrocytes, respectively, obtained through spray drying. Spherical shapes with central indentations were observed, with a size of approximately 4 µm, positive ζ potential, reddish color, and a predominant presence of carbon, oxygen, and nitrogen on their surface. Notably, the iron content of ovine and bovine erythrocytes was 2.52 and 2.24 mg of Fe/g, respectively. These values also agreed with the surface percentage characterization performed by SEM-EDS. Similar results were reported in guinea pig blood erythrocytes (3.30 mg of Fe/g) [10] and commercial bovine erythrocytes (2.49 mg of Fe/g) [42].

#### 3.1.2. Thermal Analysis

**Figure 3:**
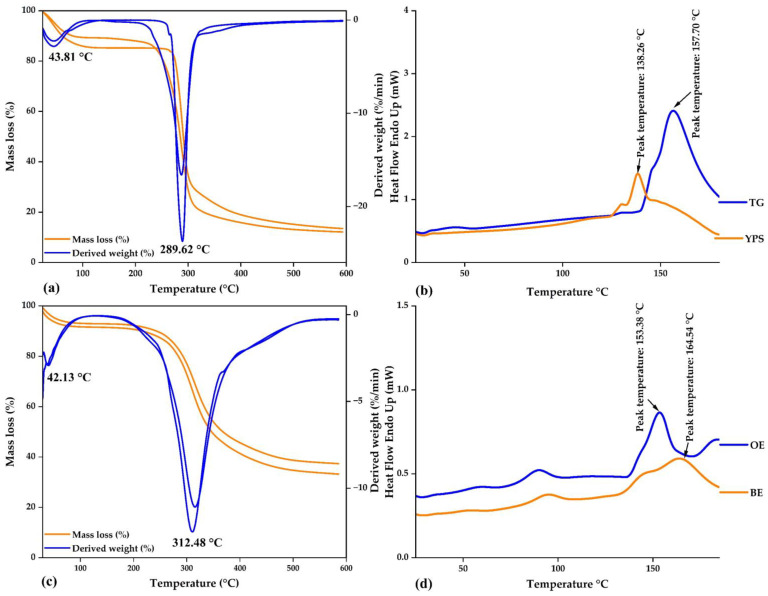
Thermal analysis: (**a**) DT and DTA curves in native potato starch and tara gum, (**b**) DSC curves in native potato starch (YPS) and tara gum (TG), (**c**) DT and DTA curves in ovine and bovine erythrocytes, and (**d**) DSC curves in ovine (OE) and bovine (BE) erythrocytes.

In Figure 3a, the thermal analysis of native potato starch from the yanapalta variety and spray-dried tara gum is presented. Both materials exhibited similar thermal behaviors, and two main events were observed. The first event occurred at a temperature of 43.81 °C, marking the initiation of hydrogen bond breaking and consequent water loss, which continued until its evaporation at around 100 °C. Additionally, other volatile components were lost during this process. The second event, observed at 289.62 °C, involved the elimination of organic compounds such as carbohydrates, proteins, lipids, and fiber, which continue degrading until reaching a temperature of 600 °C. 

Figure 3b shows the DSC analysis for both wall materials. For the yanapalta potato starch, a glass transition temperature of 138.26 °C was found, while for the spray-dried tara gum, the value was 157.70 °C. Both values are used as references to confirm the encapsulation of the cores within the wall materials. The results in both materials are consistent with the current literature, as primarily two events were observed related to the thermal degradation of biomolecules, such as carbohydrates and complex organic compounds [10,36]. DSC analysis offers valuable insights into phase transitions and changes in the molecular structure of materials. The glass transition is a crucial feature that can impact polymeric materials’ functional and encapsulation properties, holding significance for their potential applications [37,43].

On the other hand, Figure 3c,d depict the thermal analysis of spray-dried ovine and bovine erythrocytes, respectively. Two events were also observed at temperatures of 42.13 and 312.48 °C. In the first case, water loss is initiated, while in the second case, biopolymers are eliminated, ultimately resulting in the formation of ashes. The glass transition temperature was determined to be 153.38 °C for ovine erythrocytes and 164.54 °C for bovine erythrocytes. In summary, the results above provide a deeper understanding of the physical properties and their thermal behavior, holding significance for their application in various scientific and technological fields.

### 3.2. Characterization of the Microcapsules

#### 3.2.1. Physical and Chemical Properties

Table 1 presents the physical and chemical properties of microcapsules obtained from ovine and bovine erythrocytes in native potato starch and tara gum matrices. The iron content varied between 1.54 and 2.02 mg of Fe/g, with similar contents observed in microcapsules of guinea pig blood erythrocytes in native potato starch and tara gum (1.32 to 2.05 mg of Fe/g) [10] and higher contents than those of commercial bovine erythrocytes microcapsules in maltodextrin (0.77 mg of Fe/g) [42]. Iron content in the microcapsules is critical [14], particularly in food fortification [13]. Iron is essential for human health, as it plays a fundamental role in oxygen transport, DNA synthesis, and immune function [44]. The encapsulation of iron within microcapsules can confer advantages in terms of stability and bioavailability [10,42].

The percentage of total organic carbon ranged from 12.80% to 14.88%, with higher proportions of the matrices used corresponding to greater TOC contents, as carbon atoms constitute an essential part of the structure of biomolecules such as carbohydrates, proteins, lipids, and fibers [35,45,46]. The percentage quantification of TOC contents confirms the successful encapsulation of the cores within the matrices, which are rich in various biopolymers [1,14,47,48].

The encapsulation efficiency ranged from 52.94% to 85.88%, while the yield fluctuated between 50.55% and 63.40%. Encapsulation yields were similar to those reported for guinea pig erythrocyte microcapsules (47.84% and 58.73%) [10] and ferrous sulfate microcapsules (47.93% and 56.26%) [49]. On the other hand, the values exceeded those obtained in commercial bovine erythrocyte microcapsules (39% and 47%) [42].

The properties studied, which are related to the shelf-life preservation of spray-dried powders, were moisture content, ranging between 4.31% and 7.49%, and water activity, oscillating between 0.36 and 0.43. Maintaining moisture content below 5% is recommended for adequately preserving dry products, and in the case of water activity, values lower than 0.6 are advised [50,51,52,53,54]. This way, the various reaction mechanisms that lead to food deterioration are controlled. It was also observed that moisture content and water activity decrease with increased temperature and a higher proportion of encapsulants [55,56].

Likewise, variations in luminosity were observed between 52.61 and 62.59, values that increase as the proportion of matrices is increased. Regarding the color coordinate a*, values ranging between 7.00 and 11.57 were identified, and it was noted that the powders take on redder hues as the quantity of encapsulants is reduced. On the other hand, the color coordinate b* exhibited variation in the range of 18.09 to 20.55. Additionally, significant differences were observed regarding the initial color, as evidenced by ΔE*_ab_ values ranging from 5.25 to 17.84. This is attributed to non-enzymatic browning and the caramelization of carbohydrates at high temperatures [10,42].

Particle size was measured in isopropyl alcohol and using the laser diffraction technique. The values obtained ranged between 4.26 and 7.59 µm, higher than those reported for microcapsules of guinea pig erythrocytes solubilized in water and measured via the dynamic light scattering technique (817.1 and 1672.2 nm) [10]. This is attributed to the type of dispersant and the method used, with the laser diffraction technique being the most appropriate for measuring microcapsules obtained through spray drying [36,37]. On the other hand, the ζ potential presented negative values ranging from −0.11 to −3.51 mV, indicating a tendency to aggregate and precipitate due to the presence and nature of the erythrocytes used [10].

**Figure 4:**
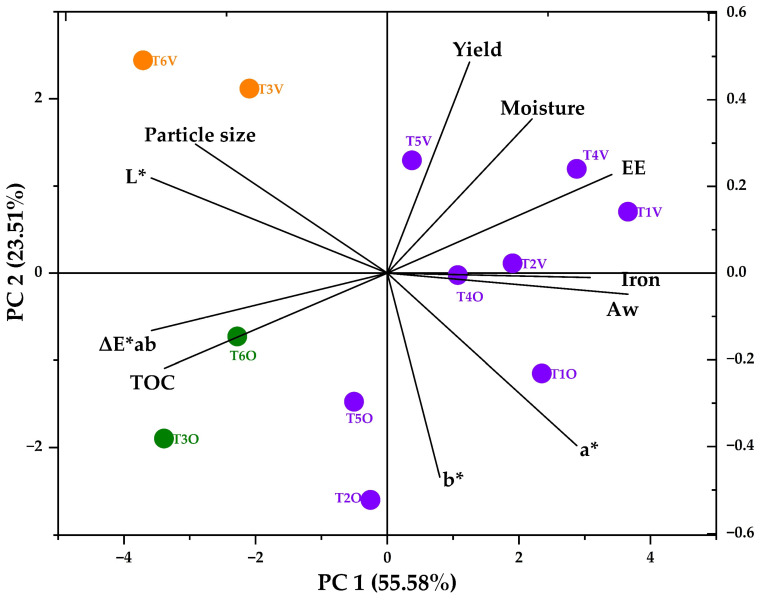
PCA study.

Figure 4 shows the principal component analysis (PCA) of the studied properties, wherein it can be observed that microcapsules T1O, T2O, T4O, T50, T1V, T2V, T4V, and T5V (in violet color) are associated with properties such as iron content, encapsulation efficiency, encapsulation yield, humidity, water activity, and color parameters a* and b*. These correlations suggest a potential interdependence among these properties, which could influence the overall performance of the microcapsules. On the other hand, microcapsules T3V and T6V (in orange color) are more linked to luminosity and particle size, indicating that these microcapsules might possess unique optical and structural characteristics that could influence their behavior, particularly in contexts involving light dispersion. In contrast, microcapsules T3O and T6O (in green color) are more related to color variations and total organic carbon.

The PCA analysis establishes relationships among complex variables, providing a comprehensive overview of interactions between diverse properties [34]. This methodology facilitates the identification of significant trends, thereby potentially aiding the design and optimization of microcapsules with specific properties [10]. PCA is a valuable tool in studying microcapsules, with potential applications within the food and pharmaceutical industries [36].

#### 3.2.2. SEM-EDS Analysis

**Figure 5:**
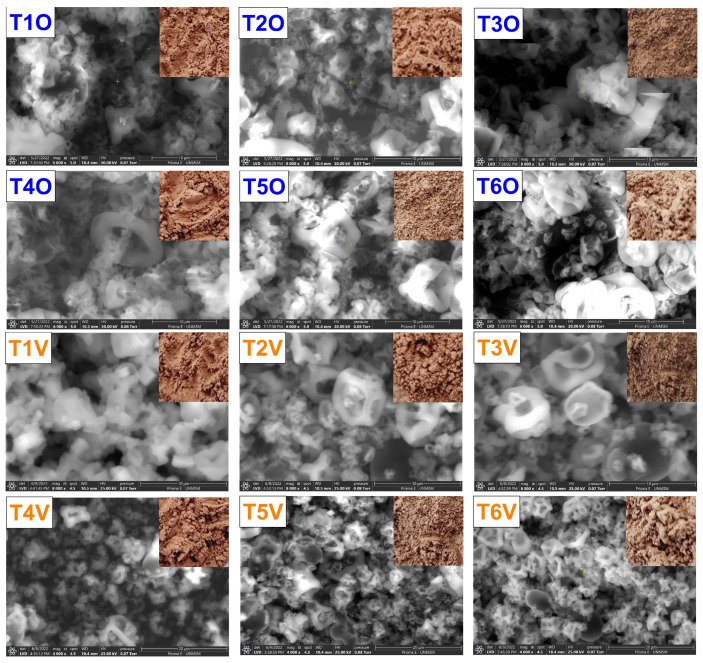
SEM analysis. Where T1O, T2O, T3O, T4O, T5O and T6O are the ovine erythrocyte microcapsules and T1V, T2V, T3V, T4V, T5V and T6V are the bovine erythrocyte microcapsules.

Figure 5 displays the SEM images of the microcapsules, revealing the formation of irregular microparticles with diverse sizes and shapes, distinctive of the spray drying process [48,57,58,59,60,61,62]. These microparticles exhibited varying dimensions and larger surface openings when a 140 °C inlet air temperature was employed. This variability appears to be influenced by feed characteristics and parameters governing the spray drying process. It is plausible that this response is linked to solvent evaporation during the drying procedure, which could have led to the contraction and loss of microcapsule sphericity [63]. The temperature increase led to a faster drying rate and the subsequent emergence of pores in the microcapsules [36]. Conversely, lower temperatures resulted in the creation of more uniform particles. However, the presence of amorphous structures in the encapsulated material is attributed to using tara gum as an encapsulating agent [10].

The results suggest that the larger size of some particles could be due to interactions between the core and matrix during microencapsulation since incorporating the core within the microcapsules may have modified the surface roughness. Iron in the microcapsules may have promoted surface collapse instead of total core retention within them [10,64].

Previous studies report on the formation of large cracks on the microparticle surface, attributing this phenomenon to the collapse of the polymeric gel network during spray drying [65]. Likewise, irradiation with electron beams by SEM can cause surface rupture of the microcapsules [66].

Surface chemical analysis of the microcapsules corroborated the existence of iron in the microcapsules, observing that an increase in the inlet temperature increased the amount of surface iron in the encapsulates, which varied between 0.1% and 0.3% (Table 2). Furthermore, it was verified that this temperature increase was associated with a larger particle size, potentially facilitating the encapsulation of a greater iron quantity. These findings align with the data obtained through ICP OES in this research.

On the other hand, carbon, oxygen, and nitrogen were observed, which could be attributed to biopolymers such as carbohydrates and proteins in the matrices and cores. The presence of these elements is consistent with previous observations of spray-dried erythrocyte microparticles [10,42]. The presence of sodium and chlorine was also observed due to the erythrocyte extraction being carried out in a saline solution. In addition, the presence of other chemical elements found in native potato starch, tara gum, and spray-dried erythrocytes was detected [10].

#### 3.2.3. FTIR Analysis

**Figure 6:**
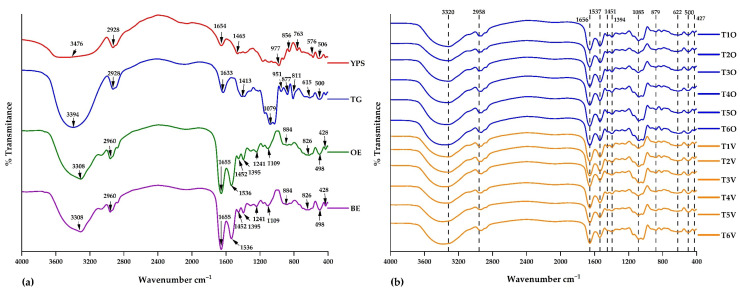
FTIR analysis: (**a**) wall materials and erythrocytes and (**b**) microcapsules.

The analysis was conducted to verify the successful microencapsulation of iron-rich cores within the utilized matrices. To achieve this, an approved methodology for acquiring and interpreting infrared spectra was followed [67]. Figure 6 displays the characteristic spectra of the examined materials: in the case of native potato starch, tara gum, and erythrocytes, intense bands within the range of 3308–3394 cm^−1^ were identified, corresponding to the stretching vibrations of the hydroxyl group. Wavenumbers with similar intensities were detected in all microcapsules (3320 cm^−1^). Furthermore, a prominent vibrational stretching band at 1079 cm^−1^ was observed in tara gum, indicative of a portion of the carboxylic acid structure. This same band was also evident in all microcapsules (1085 cm^−1^) [68,69].

Likewise, additional spectral bands present in erythrocytes were also found in the microcapsules. For instance, the wavenumber around 2960 cm^−1^, present in all microcapsules (2958 cm^−1^), corresponds to characteristic C-H stretching vibrations. Moreover, spectral bands at 1536 and 1655 cm^−1^ were identified in erythrocytes, and these bands were also observed in the encapsulated particles, with wavenumbers ranging from 1537 to 1656 cm^−1^, confirming the presence of amide functional groups I and II [42,49,70]. Bands around 622 cm^−1^ correspond to the pyranose ring of tara gum, a feature in all microcapsules [69,71,72].

The preceding results allow us to infer that the encapsulation process was successfully conducted, as the outcomes closely resembled those reported by other researchers [42,49]. Additionally, an increase in temperature from 120 °C to 140 °C induced alterations in the functional groups’ intensities [10,73].

#### 3.2.4. Thermal Analysis

**Figure 7:**
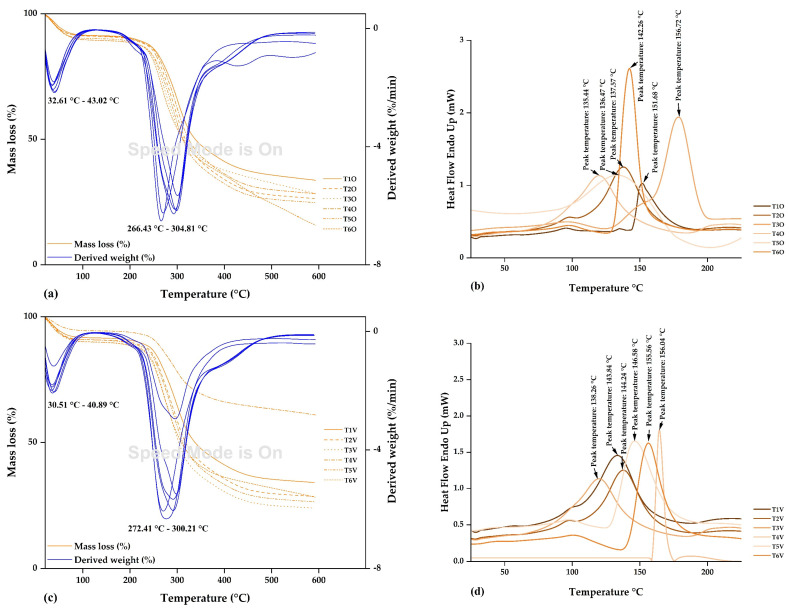
Thermal analysis: (**a**) DT and DTA curves in ovine erythrocyte microcapsules, (**b**) DSC curves in ovine erythrocyte microcapsules, (**c**) DT and DTA curves in bovine erythrocyte microcapsules, and (**d**) DSC curves in bovine erythrocyte microcapsules. Where T1O, T2O, T3O, T4O, T5O and T6O are the ovine erythrocyte microcapsules and T1V, T2V, T3V, T4V, T5V and T6V are the bovine erythrocyte microcapsules.

The TG and DTA curves in Figure 7a,c were similar in all microcapsules. A first event was observed between 30.51 °C and 43.02 °C, resulting in approximately 30% mass loss. This mass loss can be attributed to the initiation of hydrogen bond breaking in water, which continues until temperatures close to 100 °C [37,74]. A second event occurred between 266.43 °C and 304.81 °C, with a mass loss of around 90%. This pronounced degradation rate around 300 °C can be attributed to the thermal decomposition of carbohydrates. After reaching this temperature, the thermal depolymerization of biopolymers persists until complete volatilization [37,74]. According to the obtained results, it can be appreciated that the microcapsules exhibit excellent thermal stability, attributed to the use of native potato starch and tara gum as coating materials [10].

The DSC thermograms of the microcapsules are presented in Figure 7b,d, where endothermic peaks were identified at glass transition temperatures ranging from 135.44 °C to 156.72 °C. These temperatures are similar to the glass transition temperatures of native potato starch (138.26 °C) and tara gum (157.70 °C). The glass transition temperatures of the microcapsules were close to those of the used matrices. On the other hand, it is essential to mention that glass transition temperatures lower than those of the matrices would confirm the encapsulation of iron-rich erythrocyte cores [37]. These cores formed inclusive complexes with the employed encapsulants [43].

### 3.3. Iron Release

**Figure 8:**
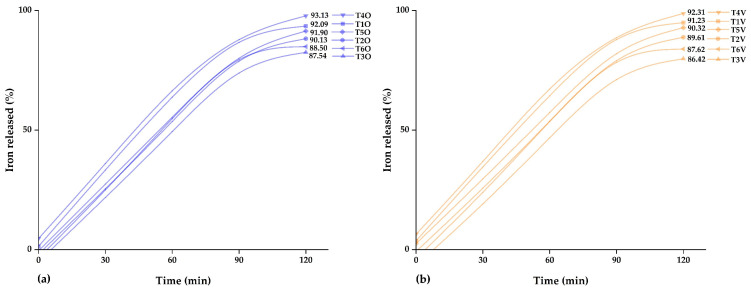
Iron released: (**a**) in microcapsules of the ovine erythrocytes and (**b**) in microcapsules of the bovine erythrocytes.

Figure 8a,b display the iron release profile from microcapsules derived from ovine and bovine erythrocytes. The outcomes indicate that, at 120 min, the spray-dried treatments T4O and T4V at 140 °C exhibited the highest iron release values, reaching 93.13% and 92.31%, respectively. It was demonstrated that an increase in the air temperature and a decrease in the encapsulating quantity result in a more pronounced iron release over time. Likewise, the other treatments also displayed considerable levels of iron release. This release is essential in bioavailability in the small intestine, as only 1 to 2 mg of iron are absorbed in this human body region, which is crucial for reducing the risk of anemia [13]. Thus, based on the developed in vitro iron release, the most prominent treatments would be T4O and T4V. The findings obtained in this study presented similarities with the results reported in studies involving iron encapsulation in matrices such as native potato starch and tara gum [10], potato starch and maltodextrin [75], chitosan and eudragit [76], eudraguard [77], and dextrin [78].

## 4. Conclusions

This study encapsulated a significant amount of heme iron extracted from ovine and bovine erythrocytes at 20% (*w*/*v*). A blend of tara gum and native potato starch was employed as a coating material at 5%, 10%, and 20% (*w*/*v*) concentrations. Encapsulation was achieved through spray drying in an aqueous environment at 120 °C and 140 °C. Consequently, elevated iron levels were achieved in erythrocytes and microcapsules, coupled with substantial in vitro bioavailability for treatments T4O and T4V and suitable physicochemical properties.

The particles exhibited micrometric dimensions and tended to aggregate in colloidal solutions. SEM-EDS analysis confirmed the presence of iron on the surface of the microcapsules. In contrast, FTIR analysis assessed the incorporation of the iron core into the polymeric matrix, substantiated by detecting functional groups within the microcapsules. Thermal analysis was also conducted, confirming the encapsulation of the cores within the matrices. In summary, the inlet temperature and the amount of coating material influenced the studied properties. Lastly, the matrix combination proved novel, and these findings pave the way for utilizing cost-effective raw materials in food fortification and combatting iron-deficiency anemia in developing nations.

## Figures and Tables

**Table 1:** Physical and chemical properties of microcapsules.

**Microcapsules O**	**T1O**		**T2O**		**T3O**		**T4O**		**T5O**		**T6O**	
Properties	$\bar{x}$ ± SD	*	$\bar{x}$ ± SD	*	$\bar{x}$ ± SD	*	$\bar{x}$ ± SD	*	$\bar{x}$ ± SD	*	$\bar{x}$ ± SD	*
Iron (mg/g)	1.99 ± 0.01	a	1.67 ± 0.01	b	1.34 ± 0.01	c	2.02 ± 0.01	d	1.87 ± 0.02	e	1.76 ± 0.02	f
TOC (%)	13.98 ± 0.01	a	14.50 ± 0.01	b	14.63 ± 0.05	b	13.78 ± 0.01	a	14.50 ± 0.03	b	14.56 ± 0.04	b
EE (%)	80.24 ± 0.21	a	66.03 ± 0.44	b	52.94 ± 0.11	c	78.72 ± 0.10	d	74.22 ± 0.73	e	69.86 ± 0.73	f
Yield (%)	53.69 ± 1.14	ab	50.55 ± 1.92	a	50.98 ± 1.91	a	56.99 ± 0.31	b	55.60 ± 0.57	ab	54.04 ± 1.46	ab
Moisture (%)	6.07 ± 0.02	a	5.07 ± 0.06	bc	4.52 ± 0.24	cd	5.27 ± 0.22	b	4.64 ± 0.12	cd	4.31 ± 0.08	e
Aw	0.43 ± 0.003	a	0.41 ± 0.004	b	0.38 ± 0.004	c	0.41 ± 0.001	b	0.40 ± 0.004	d	0.38 ± 0.002	c
L*	54.06 ± 0.02	a	55.05 ± 0.03	b	59.16 ± 0.11	c	55.95 ± 0.28	d	58.11 ± 0.22	e	60.05 ± 0.37	f
a*	11.34 ± 0.05	a	11.57 ± 0.01	a	9.63 ± 0.06	b	10.62 ± 0.18	c	10.06 ± 0.09	d	8.81 ± 0.17	e
b*	19.09 ± 0.07	a	20.55 ± 0.08	b	20.06 ± 0.06	c	18.59 ± 0.18	d	20.26 ± 0.07	bc	19.46 ± 0.17	e
ΔE*_ab_	11.41 ± 0.30	a	12.68 ± 0.31	b	16.86 ± 0.18	c	13.30 ± 0.31	b	15.82 ± 0.25	d	17.84 ± 0.67	e
Particle size (µm)	4.26 0.13	a	6.23 ± 0.05	b	6.73 ± 0.07	c	5.46 ± 0.06	d	5.60 ± 0.12	e	6.28 ± 0.10	f
ζ potential (mV)	−0.11 ± 0.16	a	−0.98 ± 0.23	b	−2.76 ± 0.91	c	−2.90 ± 0.70	d	−3.43 ± 0.47	e	−3.51 ± 0.73	f
**Microcapsules V**	**T1V**		**T2V**		**T3V**		**T4V**		**T5V**		**T6V**	
Iron (mg/g)	1.88 ± 0.01	a	1.73 ± 0.01	b	1.54 ± 0.02	c	1.93 ± 0.02	d	1.77 ± 0.01	e	1.56 ± 0.01	f
TOC (%)	13.07 ± 0.02	a	13.39 ± 0.14	ab	13.97 ± 0.04	ab	12.80 ± 0.13	a	13.65 ± 0.02	ab	14.88 ± 0.09	b
EE (%)	83.95 ± 0.22	a	78.90 ± 0.14	b	69.54 ± 0.15	c	85.88 ± 0.12	d	77.12 ± 0.10	e	68.43 ± 0.82	f
Yield (%)	60.37 ± 1.11	a	55.38 ± 1.46	b	54.93 ± 0.50	b	63.40 ± 0.50	a	62.38 ± 1.40	a	61.88 ± 0.81	a
Moisture (%)	7.49 ± 0.01	a	7.27 ± 0.08	ab	7.19 ± 0.08	b	6.22 ± 0.02	c	5.80 ± 0.07	d	5.59 ± 0.01	d
Aw	0.43 ± 0.003	a	0.43 ± 0.002	a	0.40 ± 0.003	b	0.42 ± 0.002	a	0.42 ± 0.001	c	0.36 ± 0.001	d
L*	52.61 ± 0.08	a	55.99 ± 0.14	b	61.61 ± 0.05	c	54.66 ± 0.01	d	58.20 ± 0.26	e	62.59 ± 0.08	f
a*	11.32 ± 0.08	a	9.87 ± 0.07	b	7.17 ± 0.01	c	10.51 ± 0.02	d	9.05 ± 0.11	e	7.00 ± 0.05	c
b*	19.51 ± 0.07	a	19.91 ± 0.08	b	18.34 ± 0.04	c	19.54 ± 0.02	a	19.49 ± 0.10	a	18.09 ± 0.02	d
ΔE*_ab_	5.25 ± 0.33	a	8.78 ± 0.29	b	15.17 ± 0.23	c	7.39 ± 0.24	d	11.17 ± 0.47	e	16.17 ± 0.31	f
Particle size (µm)	5.52 ± 0.03	a	5.54 ± 0.02	b	7.34 ± 0.21	c	5.97 ± 0.11	d	6.92 ± 0.09	e	7.59 ± 0.15	f
ζ potential (mV)	−0.30 ± 0.38	a	−0.40 ± 0.05	b	−1.46 ± 0.61	c	−0.51 ± 0.12	d	−1.10 ± 0.06	e	−1.89 ± 0.16	f

Where $\bar{x}$ is the arithmetic mean and SD is the standard deviation. * Different letters indicate significant difference per row evaluated through at 5% significance, for n = 3.

**Table 2:** Surface chemical analysis of microcapsules via EDS.

Element	Weight%											
	T1O	T2O	T3O	T4O	T5O	T6O	T1V	T2V	T3V	T4V	T5V	T6V
C	46.1%	43.3%	39.8%	43.5%	41.2%	39.7%	41.6%	39.8%	41.7%	39.3%	39.1%	41.7%
O	25.8%	33.8%	37.7%	33.5%	35.7%	38.6%	24.1%	33.4%	35.1%	25.8%	37.0%	34.4%
N	12.3%	10.1%	9.8%	10.1%	9.1%	8.6%	7.7%	7.5%	5.8%	7.6%	7.8%	6.1%
Na	8.3%	7.0%	6.4%	6.2%	7.0%	6.7%	15.3%	9.6%	8.8%	14.5%	7.8%	9.4%
Cl	6.7%	4.9%	5.4%	6.0%	6.1%	5.3%	10.4%	8.9%	7.8%	11.9%	7.5%	7.3%
S	0.5%	0.6%	0.6%	0.3%	0.4%	0.7%	0.4%	0.3%	0.3%	0.4%	0.3%	0.4%
Fe	0.1%	0.1%	0.1%	0.2%	0.2%	0.2%	0.2%	0.2%	0.2%	0.3%	0.3%	0.3%
P	0.1%	0.1%	0.1%	0.1%	0.1%	0.1%	0.1%	0.1%	0.1%	0.1%	0.1%	0.1%
K	0.1%	0.1%	0.1%	0.1%	0.2%	0.1%	0.2%	0.2%	0.2%	0.1%	0.1%	0.3%

## Data Availability

They are available in the same article.
